# Put a tiger in your tank: the polyclad flatworm *Maritigrella crozieri* as a proposed model for evo-devo

**DOI:** 10.1186/2041-9139-4-29

**Published:** 2013-10-09

**Authors:** François Lapraz, Kate A Rawlinson, Johannes Girstmair, Bartłomiej Tomiczek, Jürgen Berger, Gáspár Jékely, Maximilian J Telford, Bernhard Egger

**Affiliations:** 1 Department of Genetics, Evolution and Environment, University College London, London, UK; 2 Department of Biology, Dalhousie University, Halifax, NS, Canada; 3 Department of Evolutionary Developmental Biology, Institute of Zoology, University of Innsbruck, Innsbruck, Austria; 4 Max Planck Institute for Developmental Biology, Tübingen, Germany

**Keywords:** Evolutionary and developmental biology, Larvae, Neuropeptides, Planarians, Polyclad flatworms, Regeneration, Spiralians, Stem cells, Transcriptome, Turbellarians

## Abstract

Polyclad flatworms are an early branching clade within the rhabditophoran Platyhelminthes. They provide an interesting system with which to explore the evolution of development within Platyhelminthes and amongst Spiralia (Lophotrochozoa). Unlike most other flatworms, polyclads undergo spiral cleavage (similar to that seen in some other spiralian taxa), they are the only free-living flatworms where development via a larval stage occurs, and they are the only flatworms in which embryos can be reared outside of their protective egg case, enabling embryonic manipulations. Past work has focused on comparing early cleavage patterns and larval anatomy between polyclads and other spiralians. We have selected *Maritigrella crozieri*, the tiger flatworm, as a suitable polyclad species for developmental studies, because it is abundant and large in size compared to other species. These characteristics have facilitated the generation of a transcriptome from embryonic and larval material and are enabling us to develop methods for gene expression analysis and immunofluorescence techniques. Here we give an overview of *M. crozieri* and its development, we highlight the advantages and current limitations of this animal as a potential evo-devo model and discuss current lines of research.

## Review

Platyhelminthes, or flatworms, are a group of soft-bodied, usually hermaphroditic invertebrates especially renowned for their neoblast stem cell system and their pronounced ability to regenerate [1,2]. They are members of the Spiralia, and, with recent evidence supporting the exclusion of the Acoelomorpha and Xenoturbellida (see, for example, [3]), they comprise two monophyletic groups: the Catenulida and the Rhabditophora [4,5], with the latter including the Polycladida.

The Polycladida are among the earliest branching Platyhelminthes [6-9]. They sport a highly branched gut (the name-giving feature of the Polycladida), and they have retained certain developmental characteristics common to other spiralian taxa, such as endolecithal eggs and (at least partially) spiral cleavage [10-12]. Embryonic development features a prominent mesentoblast and epibolic gastrulation. Whereas all other free-living flatworms are direct developers, both direct and indirect development is known in polyclads. Indirect development typically features a spherical, usually eight-lobed, three-eyed Müller’s larva. Other larval forms include a four-lobed, two-eyed Goette’s larva and a dorsoventrally flattened, eight-lobed, twelve-eyed Kato’s larva.

All cotylean species studied feature a Müller’s larva with eight lobes and three eyes, with three listed modifications involving intracapsular metamorphosis and sometimes a reduced number of lobes and eyes. In acotyleans, predominantly direct development occurs, but Müller’s larvae, Goette’s larvae with four lobes and usually two eyes, and dorsoventrally flattened Kato’s larvae with eight lobes and twelve eyes are also known (summarized in [8], Table 1). In several cases, more than one developmental type has been described in a genus, sometimes even in the same species. This may be partly attributed to the difficulty of correct species determination in many acotyleans, which is also reflected in frequent taxonomic rearrangements, such as in the cases of *Hoploplana inquilina* (formerly *Planocera inquilina*), *Stylochus ellipticus* (formerly *Planocera elliptica*) and *Stylochoplana maculata* (formerly *Stylochus maculatus*).

**Table 1:** Developmental types in polyclads

**Suborder**	**Superfamily/family**	**Genus/species**	**Development/larva type**
Cotylea			
	Euryleptoidea/Euryleptidae	*Maritigrella crozieri*	Müller’s larva [12-14]
	Euryleptoidea/Prosthiostomidae	*Amakusaplana acroporae*	Intracapsular Müller’s larva [15]
	Pseudocerotoidea/Pericelidae	*Pericelis cata*	Intracapsular/reduced Müller’s larva [16]
	Pseudocerotoidea/Boniniidae	*Boninia divae*	Reduced Müller’s larva [16]
Acotylea			
	Leptoplanoidea/Notoplanidae	Notoplana	
		*Notoplana alcinoi*	Direct development [17]
		*Notoplana australis*	Goette’s larva [18]
		*Notoplana delicata*	Direct development [19]
	Leptoplanoidea/Leptoplanidae	Hoploplana	
		*Hoploplana inquilina*	Müller’s larva [10,20]
		*Hoploplana villosa*	Direct development [19]
	Leptoplanoidea/Stylochoplanidae	Stylochoplana	
		*Stylochoplana agilis*	Direct development [B Egger, unpublished observations]
		*Stylochoplana maculata*	Müller’s larva [21] after [22]/direct development [23]
		*Stylochoplana parasitica*	Direct development [19]
	Stylochoidea/Stylochidae	Stylochus	
		*Stylochus (Imogine) aomori*	Goette’s larva [19]
		*Stylochus (Imogine) mcgrathi*	Goette’s larva [24]
		*Stylochus (Imogine) mediterraneus*	Goette’s larva [25]
		*Stylochus (Imogine) uniporus*	Goette’s larva [19]
		*Stylochus (Imogine) zebra*	Direct development [26,27]
		*Stylochus (Stylochus) flevensis*	Goette’s larva [28]
		*Stylochus (Stylochus) frontalis*	Direct development [29]
		*Stylochus (Stylochus) neapolitanus*	Direct development [21]
		*Stylochus (Stylochus) pilidium*	Goette’s larva [21]
		*Stylochopsis ellipticus*	Goette’s larva [21] after [30]/Müller’s larva [31]
		*Stylochus luteus*	Müller’s larva [21] after [32]
	Stylochoidea/Planoceridae	Planocera	
		*Planocera multitentaculata*	Müller’s larva [19]
		*Planocera reticulata*	Intracapsular larva [19]/Kato’s larva [33,34]/direct development [35]

Adult polyclads are extremely dorsoventrally flattened, with body lengths typically in the centimetre range. They are subdivided into two groups characterized by the presence (Cotylea) or absence (Acotylea) of a cup-shaped ventral sucker located along the midline of the body and posterior to the genital openings [21,36]. Although larval and juvenile stages may be found swimming in the water column, adults are usually found on the substrate [37]. Polyclads are capable of regeneration, but that does not include the regeneration of the head, with the exception of the acotylean *Cestoplana*, in which pieces cut just posterior of the brain are able to regenerate the brain [38].

Herein we highlight the polyclad *Maritigrella crozieri* as a candidate model for evo-devo. We review past work done on this species, give a preview of new experimental data and discuss the most relevant scientific topics and future directions.

### *Maritigrella crozieri*, a polyclad species for evo-devo

Despite the Lophotrochozoa being one of the three major branches of bilaterians, very few representatives of the Lophotrochozoa are found amongst classical experimental models. Emerging new model molluscs, annelids and platyhelminths have been pushed forward recently to fill this taxonomic gap [39]. Among these, the principal platyhelminth models are triclads, which, despite being valuable models for stem cell and regeneration biology, have a derived mode of development, making comparative studies difficult ([40]; see also review in [8]).

We have chosen *M. crozieri* as a suitable polyclad representative for evolutionary and developmental studies. The major advantages of this species are its ease of collection, its readily observable spiral cleavage, its biphasic life cycle with an eight-lobed Müller’s larva and its large size with many eggs, which can be obtained and raised without eggshells.

### Description of *Maritigrella crozieri*

**Figure 1:**
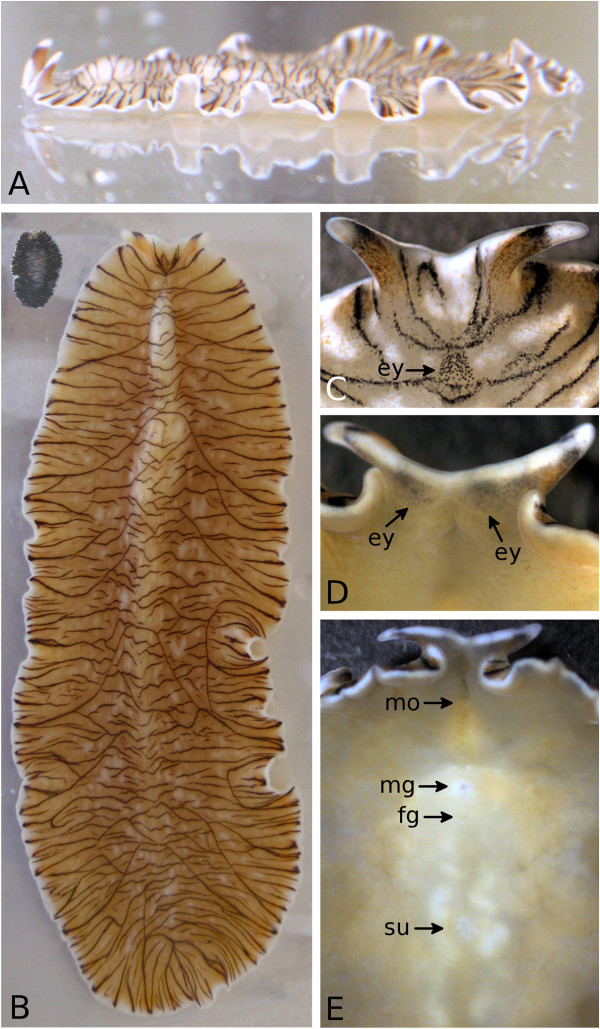
**Habitus of adult*Maritigrella crozieri*. (A)** Side view of a specimen gliding on the wall of a glass tank with undulating motions of the margins, illustrating the dorsoventral flatness. **(B)** Largest specimen found so far, about 56 mm in length in quiet position. Inset at top left is another specimen after about 4 months of starvation, with a measured length of 5.6 mm, one-tenth that of the large specimen. Note the darker colour of the starved specimen. **(C)** Dorsal view of the anterior part, with elongated tentacles and two merging patches of cerebral eyes (ey). **(D)** Ventral view of the anterior part with immersed tentacular eyes (ey). **(E)** Ventral view with mouth opening (mo) below a slitlike fold, the male genital opening (mg), the female genital opening (fg) and the sucker (su). Parts **(C)** through **(E)** show the same specimen.

**Figure 2:**
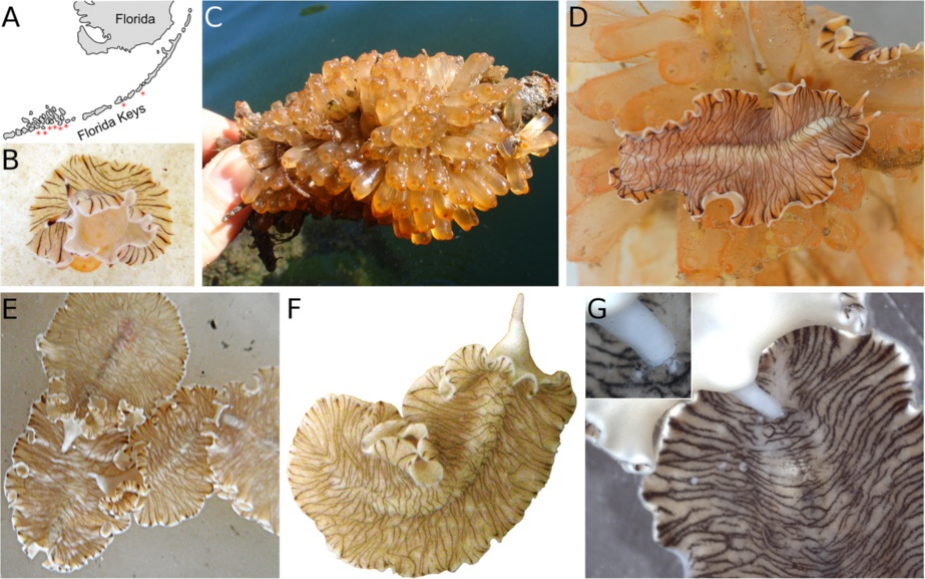
**Sampling sites, feeding and copulation of*Maritigrella crozieri*. (A)** Schematised map of the Florida Keys. Red asterisks indicate collection sites. **(B)** Adult *M. crozieri* wrapped around and feeding on a zooid of the ascidian *Ecteinascidia turbinata*. **(C)** A colony of *E. turbinata* growing on a mangrove root, lifted from the water. **(D)** Adult *M. crozieri* gliding on a colony of *E. turbinata* underwater. Parts **(E)** through **(G)** illustrate copulation. **(E)** Two adult *M. crozieri* with extended male copulatory organ (mco) gliding onto a third animal, with another specimen approaching from the right. **(F)** Single adult with fully extended mco; anterior is up. **(G)** Sperm deposition (white spots) on the dorsal side of another adult. Inset shows detail of the mco with two sperm deposits (see also Additional file 1).

*M. crozieri* was first mentioned in the literature as *Pseudoceros* sp. [41], and the formal species description gives the same species the name *Pseudoceros crozieri*[42]. Newman *et al*. [43] reclassified the species as a euryleptid belonging to the genus *Maritigrella*[44], based on the anterior tubular pharynx and eye clusters at the base of the elongate tentacles (Figures 1 and 2).

On the dorsal surface, it has an irregular pattern of black stripes on a white to orange background that inspired the name for the genus [44] (Figures 1A through 1C, 2B and 2D through 2G). The eyes are located in two broad wedges, merging dorsal to the brain (cerebral eyes) and at both the ventral and dorsal bases of the tentacles (tentacular eyes, Figure 1C and 1D). We have counted more than 90 cerebral eyes in an individual (Figure 1C), which is slightly higher than the number (about 70) reported by Newman *et al*. [43]. The animal’s maximum reported length was up to 30 mm [42] and 31.3 ± 2.7 mm (*n* = 20) [12]. We have since found several significantly larger individuals, with the greatest measured length being 56 mm (Figure 1B).

**Figure 3:**
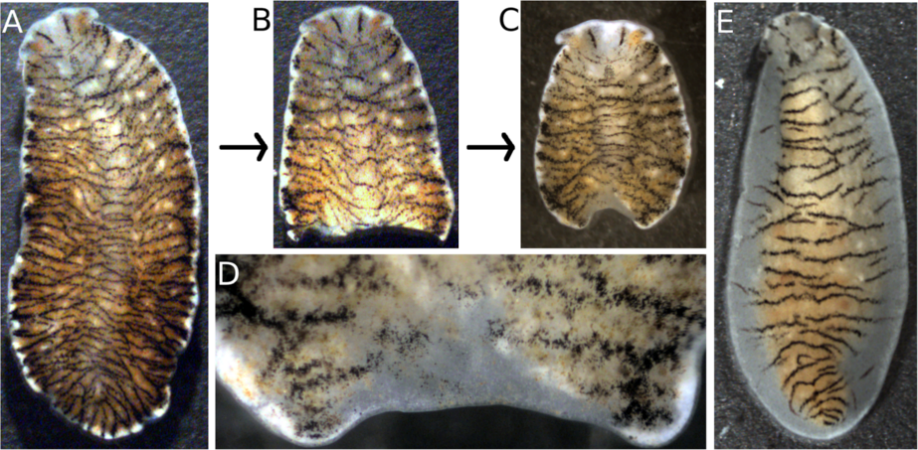
**Regeneration of adult*Maritigrella crozieri*.(A)** through **(D)** show a series of images of the same individual. **(A)** Intact adult. **(B)** Anterior part of the animal cut transversally in two parts. **(C)** Anterior part after 16 days of regeneration. The blastema is visible as a white crescent in the posterior end. **(D)** Detail of the blastema shown in **(C)**. **(E)** Adult animal found in the culture that regenerated the whole margin (white) from the orange core. The black stripes have already extended to the regenerated margins.

Externally, *M. crozieri* can be distinguished from its mostly stripy congeners by its distinct dorsal pigmentation, its size and its geographical distribution (see [43-46]). We amend the species description [43] to “margin with irregular banding of opaque white, sometimes with a semitransparent outer white band (Figure 2G) and orange pigment speckles (Figure 3D),” as we have never observed an outer orange band in our specimens, nor have we identified it in the figures published by Newman *et al*. [43]. The base of the tentacles is orange, followed by a black stripe and a white tip ([43], Figure 1C).

The male genital opening is anterior to the female opening, with the sucker being situated behind them ([43], Figure 1E). Whereas Hyman [42] stated that “there is no stylet” in later works the stylet (the sclerotised tip of the male copulatory organ) was found to be present and to be about 130 μm long [43]. Insemination has been reported to occur hypodermically by stabbing [43]. In our observations, animals were not stabbing each other, but rather gently depositing sperm on the dorsal surface of their partners with their stylet (Figure 2E through 2G; see also Additional file 1). Histological sections of the spermatophores on or in the epidermis are required for ascertaining dermal impregnation instead of hypodermic insemination [36].

### *Maritigrella crozieri* collection and prey preference

The type locality of *M. crozieri* is Bermuda [41,42], and animals have subsequently been collected off mainland Florida and the Florida Keys [12,13,43,47]. All our sampling efforts were concentrated on the Florida Keys, and we found animals (from west to east) on Sugarloaf Key, Cudjoe Key, Summerland Key, Ramrod Key, Big Pine Key, No Name Key, Long Key and Upper Matecumbe Key (Figure 2A). While most animals could be found on the ascidian *Ecteinascidia turbinata* (Figure 2B through 2D), specimens of *M. crozieri* were occasionally encountered on mangrove roots without *Ecteinascidia* or on the shallow ocean floor.

*M. crozieri* shows a strong preference for the orange ascidian *E. turbinata* as a food source [41,43]. We occasionally found animals with a purple gut instead of an orange gut, indicating other prey items. Crozier [41] identified *Ascidia curvata* and *Phallusia nigra* (formerly *Ascidia atra*) as other possible prey for *M. crozieri* and speculated that the food specificity of adult specimens of *M. crozieri* may be an acquired taste of the juvenile, depending on which ascidian species the juvenile had settled. Newman *et al*. [43], on the other hand, claim that *M. crozieri* feeds exclusively on *E. turbinata*.

**Figure 4:**
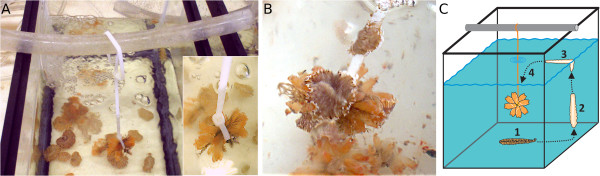
**Swimming experiment. (A)** Mimicking a free-hanging mangrove root with attached ascidians (*Ecteinascidia turbinata*), a colony of ascidians is lowered into a tank (without touching the bottom; see inset) filled with water and adult *M. crozieri*. **(B)** Several specimens of *M. crozieri* have reached the dangling ascidians. The flatworms are not able to swim directly towards the food through the water. Instead, they glide up the glass wall and move under the water surface using adhesive surface forces and fluid resistance to prevent sinking and let themselves drop onto the ascidians when floating directly above them. **(C)** Schematic showing four steps of a polyclad reaching the dangling ascidians, starting from the bottom of the tank, then gliding up the glass wall and under the water surface.

Our observations suggest that adult *M. crozieri* are unable to swim. To find out how adult *M. crozieri* can reach ascidian colonies on dangling mangrove roots, we suspended *E. turbinata* colonies on string in the centre of saltwater tanks (Figure 4; see Additional file 2). We observed individuals moving up the side of the tank and floating under the water surface before curling up to break the water surface tension and descending down onto the ascidians. We hypothesise that the Müller’s larvae of *M. crozieri* undergo metamorphosis before they reach the ascidians and that metamorphosed juveniles could use the same technique as adults to reach the *E. turbinata* prey.

### Laboratory cultures and development

Specimens of *M. crozieri* were able to survive up to 143 days without feeding, shrinking significantly during this time of starvation (Figure 1B, inset) at room temperature in 3.5% artificial seawater (hw-Meersalz professional and hw-Marinemix professional; Wiegandt GmbH, Krefeld, Germany).

The egg-laying period in the laboratory lasts for more than 3 months (107 days), showing that animals under ongoing starvation are still able to produce and lay eggs. The great majority of eggs are laid during the first 2 months of captivity. Gravid *M. crozieri* lay eggs directly on the tunic of *E. turbinata* in the wild and on the side of their container, or even under the water surface, when in captivity. Each embryo is contained within a thick spherical capsule, which collectively form a compact monolayer (egg plate) of 50 to 1,000 capsules. Importantly for experimental manipulation of the embryos, puncturing the paired uteri of gravid adults with a dissecting needle releases viable fertilised eggs devoid of their capsules (see [48]). If raised in a gelatin-coated petri dish in filtered seawater treated with antibiotics, these naked eggs develop in the same way as their encapsulated counterparts [12].

**Figure 5:**
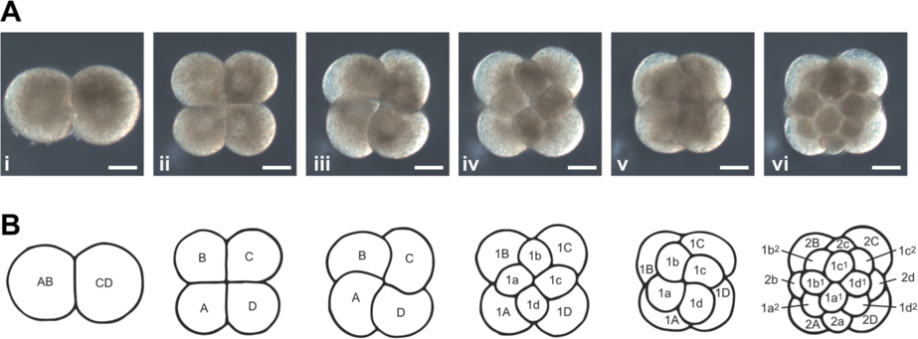
**First cleavages during early development of*Maritigrella crozieri*.** Fertilised eggs devoid of an eggshell were collected using the method of Boyer [48], then coverslipped and observed under a microscope in a room at 27°C. (Ai) through (Avi) show extended focus snapshots of animal views of the 4 four first cleavage stages from a 4D time-lapse movie using Helicon Focus software (HeliconSoft, Kharkov, Ukraine). (Ai) Two-cell stage. (Aii) Four-cell stage. (Aiii) Late four-cell stage showing dextral torsion of the four blastomeres. (Aiv) Eight-cell stage after division of the first quartet micromeres. (Av) Late eight-cell stage showing sinistral torsion of the blastomeres. (Avi) Sixteen-cell stage after division of the second-quartet micromeres. **(B)** Diagram corresponding to the cleavage stages shown in the part **(A)** images. Blastomere nomenclature according to Surface [10], with quadrants arbitrarily assigned in species with equal cleavage. Scale bars represent 50 μm.

**Figure 6:**
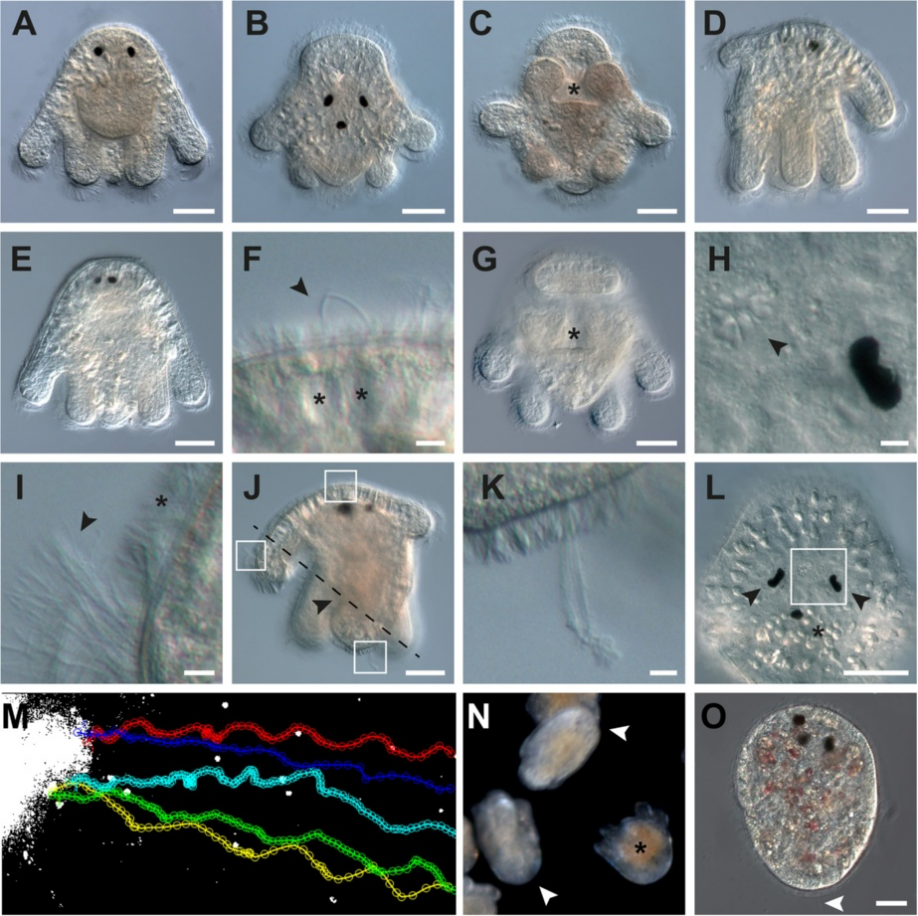
**Morphology and behaviour of*Maritigrella crozieri*larvae.** Images **(A)** through **(L)** show larvae 1 day after hatching. **(A)** through **(E)**, **(H)** and **(L)** are extended-focus images produced using Helicon Focus software (HeliconSoft, Kharkov, Ukraine). **(F)**, **(G)** and **(I)** through **(K)** are single-focus images. **(A)** Ventral view. **(B)** Anterior view. **(C)** Posterior view showing the mouth opening (asterisk). **(D)** Right-side view. **(E)** Dorsal view. **(F)** Long cilia (arrow), and large cells (asterisk), are found in the apical tuft region. **(G)** View of the focal plane illustrated by a dashed line in **(J)** showing the mouth opening (asterisk). **(H)** A rosette of cells surround the long cilia of the apical organ (arrow). **(I)** Short cilia cover the epidermis (asterisk), while long cilia cover the ciliary band cells (arrow). **(J)** Leftside view showing the mouth opening (arrow). Top, left and bottom white squares are enlarged in **(F)**, **(J)** and **(K)**, respectively. **(K)** Long cilia of the posterior tuft. **(L)** Anterior view. Epithelium with rhabdites surround the apical organ. White square is enlarged in **(H)**. Cerebral and epidermal eyes are marked with arrows and an asterisk, respectively. **(M)** Trajectories of larvae swimming in a right-handed helical motion towards a light source. Trajectories were deduced from a movie of 2-day-old larvae swimming in an embryo dish (Supplementary movies 3 and 4) and using the MTrackJ plugin [50] of Fiji software (http://www.fiji.sc). **(N)** Larval shapes vary from roundish (asterisk) to elongated (arrows) due to muscular contractions. Yolk granules are marked with an asterisk. **(O)** Left side view of a 5-week-old larva. Note the decreased size, presence of the posterior tuft (arrowhead) and the resorption of lobes. Scale bars are 50 μm in **(A)** through **(E)**, **(G)**, **(J)**, **(L)** and **(O)** and 10 μm in **(F)**, **(H)**, **(I)** and **(K)**.

**Figure 7:**
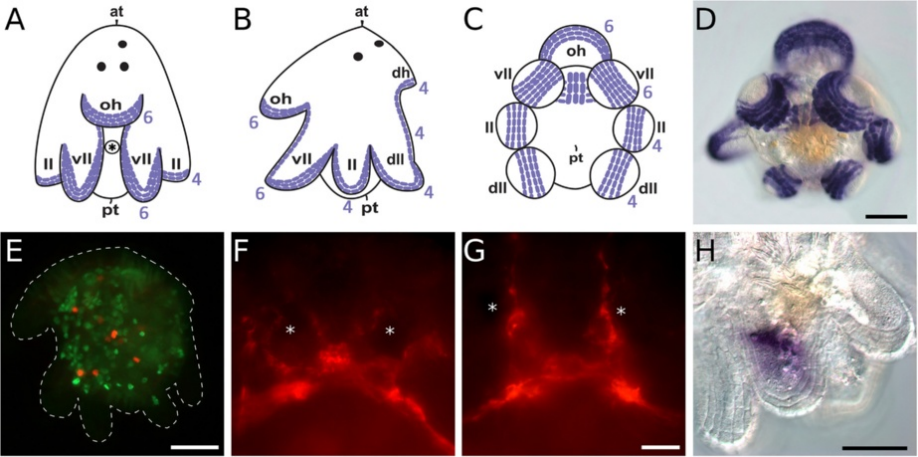
**Ciliary band and antibody staining in*Maritigrella*larvae. (A)** through **(C)** Schematic drawings of 1-day-old larvae (from left to right: ventral view, lateral view and posterior view, respectively) featuring the ciliary band cells in purple. Numbers are related to rows of ciliary band cells. **(D)** Posterior view of fixed larva with stained ciliary band cells. **(E)** Larva after a 20-h bromodeoxyuridine (BrdU) pulse: S-phase-labelled cells are shown in green using an antibody against BrdU, and mitoses are shown in red using an antibody against phosphorylated histone H3. Dashed outline: ventral is left and anterior is up. **(F)** through **(G)** Larval brain seen from the anterodorsal side. Staining with antibodies against the neuropeptides AVRLIRLamide **(F)** and GVWSNDPWamide **(G)**. Asterisks demarcate the position of the cerebral eyes. **(H)** Ciliary band cells visualised by differential interference contrast imaging. Purple in **(D)** and **(H)** is unspecific staining. Scale bars are 50 μm in **(D)** and **(E)** and 10 μm in **(G)** and **(H)**. Scale bar in **(F)** identical to that in **(G)**.

**Figure 8:**
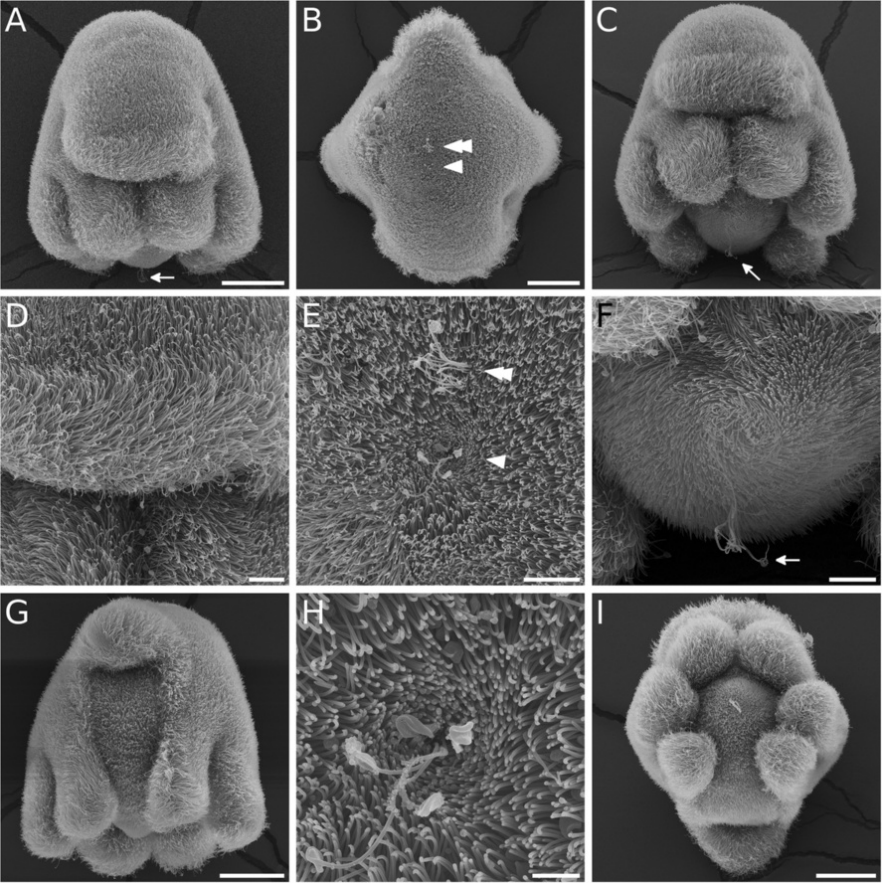
**Scanning electron photomicrographs of 1-day-old Müller’s larvae of*Maritigrella crozieri*. (A)** and **(D)** Anteroventral views. Note longer cilia at the tip of the oral hood and the lobes. **(B)**, **(E)** and **(H)** Anterior views. Dorsal side is up, ventral is down. The apical tuft is in the centre (arrowhead), and a bundle of longer cilia (double-arrowhead) is located dorsally to the apical tuft. **(C)** and **(F)** Ventral views. **(G)** Dorsal view showing the dorsal lobe at the top and pairs of dorsolateral, lateral and ventrolateral lobes. **(I)** Posterior view showing the tip of the oral hood on top, followed by pairs of ventrolateral, lateral and dorsolateral lobes and at the bottom the dorsal hood. All arrows point to the posterior tuft. Scale bars are 50 μm in **(A)**, **(B)**, **(G)** and **(I)**; 10 μm in **(D)** and **(F)**; and 3 μm in **(H)**. Scale bars in **(A)** and **(C)** are identical.

Embryonic development of *M. crozieri* has been described in detail by Rawlinson [12]. First divisions show a typical quartet spiral cleavage mode (Figure 5) with equal blastomere size. Müller’s larvae hatch after about 8 days with three eyes (two cerebral eyes and one epidermal eye) and eight lobes (Figures 6, 7 and 8). The epidermis is completely covered by short cilia and includes rhabdite cells towards the anterior and posterior poles (Figure 6L). Larvae show a well-developed ciliary band with longer cilia (Figures 6A through 6E, 6G, 6I, 7A through 7D, 7H, 8A through 8D, 8G and 8I) that follows along the eight protruding lobes. There is an apical organ with long cilia surrounded by a ring of flask-shaped glandular or neuronal cells [12] (Figures 6F, 6H, 6J, 6L, 8B, 8E and 8H). Very close to the apical organ, but shifted slightly dorsally, a bundle of longer cilia can be seen (Figure 8B and 8E). These cilia have not yet been described, and their significance is unknown so far. There is also a caudal cilium (posterior tuft) lying on the dorsal side of the posterior pole (Figures 6J, 6K, 6O, 8A, 8C, 8F and 8I). The mouth is on the ventral side (Figure 6C, 6G and 6J) and forms the single opening into the larva’s unbranched and ciliated blind gut [12]. Although yolk granules are still present in endodermal cells when they hatch (Figure 6N), the larvae of *M. crozieri* have been reported to feed on phytoplankton [14]. Like the trochophore larvae of the annelid *Platynereis*[49], larvae of *Maritigrella* swim in a right-handed helical motion in the water column and are typically positively phototactic [12,13] (Figure 6M, Additional file 3 and Additional file 4). Larvae kept at 20°C survived for more than 5 weeks (37 days) after hatching, in contrast to the findings of Johnson and Forward [13], who reported 3 weeks as the end of the larval life span of *M. crozieri*. During starvation, the larval lobes get resorbed continuously, so that the larvae eventually resemble a slightly elongated sphere (Figure 6O).

### Genome and transcriptome

The haploid genome size (1C) of *M. crozieri* is estimated to be about 2.5 Gb (2,511.2 ± 35.8 Mb; *n* = 3) by flow cytometry (J Spencer Johnston, personal communication), providing the first genome size information for a polyclad flatworm. Diploid genome sizes of 38 free-living flatworms were shown to have a considerable range between about 0.1 and 40 Gb, with an average of about 5 Gb [51], which corresponds very closely to *M. crozieri*’s diploid genome size.

**Figure 9:**
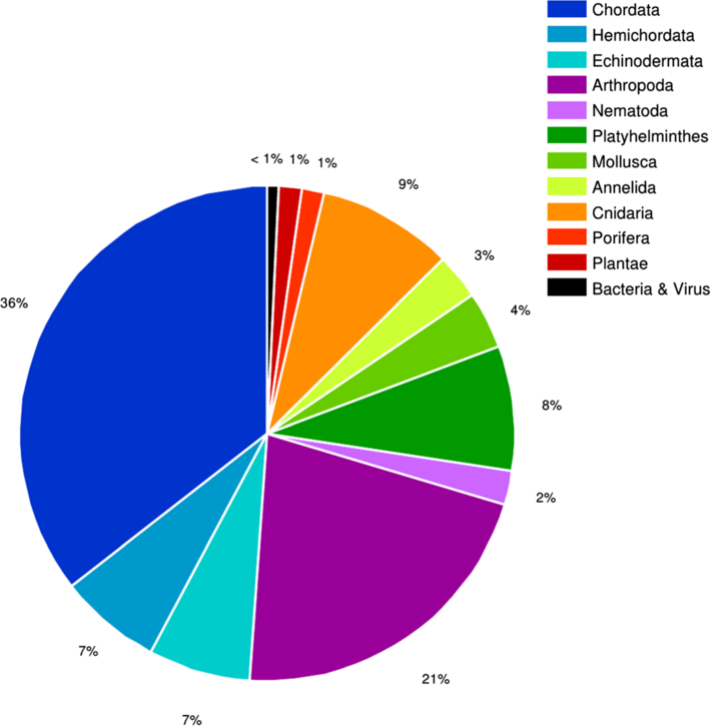
**Pie chart showing the species distribution of unigenes top tblastn results against the nonredundant protein database with a cutoff** ***E*** **value <1e**^**-6**^**.**

We have extracted and sequenced RNA from embryonic and larval stages of *M. crozieri*. Using Trinity RNA-seq *de novo* assembly software [52], clustering of all contigs larger than 300 nucleotides resulted in 72,924 unigenes from among 128,196 isoforms (56.9%), and clustering of all contigs larger than 500 nucleotides resulted in 34,745 unigenes from among 77,383 isoforms (44.9%) (Table 2). Isoforms represent all valid transcripts (above a certain arbitrary threshold), and unigenes gather different isoforms (or splicing forms) of a gene into a cluster [53]. The average length of all contigs is 807 bp, and the median length is 351 bp (Table 2), which demonstrates the high quality of the assembly. In a random subset of sequences, less than 1% of significant best BLAST hits were bacterial and viral sequences (Figure 9), which indicates a low level of contamination by these groups in the transcriptome. Only 8% of the sequences from the *M. crozieri* transcriptome recognize other flatworm sequences as the best BLAST hit (Figure 9), reflecting the necessity of further bioinformatics research within Platyhelminthes.

**Table 2:** **General properties of the** ***Maritigrella crozieri*** **transcriptome assembly produced using the Trinity assembly tool for** ***de novo*** **reconstruction of transcriptome sequences from RNA-seq data**

**Parameter**	**Data**
Number of contigs	216,151
Total size of contigs	174,358,739
Longest contig	23,763
Shortest contig	201
Number of contigs >500 nucleotides	77,383 (35.80%)
Number of contigs >1,000	44,206 (20.50%)
Number of contigs >10,000	399 (0.20%)
Number of contigs >100,000	0 (0%)
Mean contig size	807
Median contig size	351
Contig %A	29.44
Contig %C	20.51
Contig %G	20.68
Contig %T	29.37
Contig %N	0.00

### Regeneration and stem cells

The regenerative capacity of some flatworms has been known for two centuries [54] and is reliant on the neoblast stem cell system. Some flatworms can readily regenerate a head when cut into 100 pieces [55] or regenerate to a complete organism from only 1,500 cells [56]. However, not all flatworms are able to regenerate all missing body parts or even to regenerate at all [2]. The “polyclad rule for regeneration” was postulated as the ability to regenerate all parts of the body but the brain [57]. This is true for all polyclads studied so far, with the notable exception of *Cestoplana*, which is able to regenerate its head from the posterior fragment when amputated just posterior to the brain [38]. Although more than 10 acotylean polyclads have been observed to regenerate [2], only a single cotylean species, *Thysanozoon brocchii*, has been the subject of published regeneration studies [58,59]. Herein we can report that *M. crozieri* (*n* = 3) is able to regenerate both laterally and posteriorly at room temperature (Figure 3), although regeneration in small animals that have not fed for weeks or months is slow and extensive regeneration studies are still outstanding.

Neoblasts are totipotent stem cells and the only proliferating cells in adult rhabditophoran flatworms [60,61]. In the Rhabditophora, neoblasts are located exclusively in the mesenchymal space and at the base of the gastrodermis, but they are conspicuously absent in the epidermis [62]. Migration of stem cells into the epidermis has been demonstrated for juvenile polyclads [62,63]. Interestingly, in late embryos of *Notocomplana humilis* and *Cycloporus japonicus*, and even in the Müller’s larvae of the latter, mitoses were detected in the epidermis [63]. In *M. crozieri* larvae, the first Müller’s larvae with successful bromodeoxyuridine (BrdU) labelling following the protocol given by Egger *et al*. [62] using 20-h BrdU incubation time, we could not detect proliferating cells (S-phase or mitotic) within the epidermis (see Figure 7E).

Regeneration in flatworms and stem cell research on them so far have been focused mainly on triclads and *Macrostomum*[64], but they are interesting topics to study in polyclads. In particular, the absence (as in triclads [65]) or presence (as in Macrostomum [66]) of proliferating cells within the regeneration blastema will help determine the ancestral mode of tissue repair in flatworms. Large polyclads such as *M. crozieri* are amenable to microsurgery, thereby allowing us to explore more precisely the limits of their regenerative capacity.

### Hypotheses on phylogenetic relationships and character evolution

Among the metazoan phyla, many diverse groups have a biphasic life cycle in which they pass through a pelagic larval stage that gives rise to a benthic adult animal through metamorphosis. The presence of such larvae is patchily distributed, however, raising the question of their evolutionary origin. The biphasic life cycle could be an ancient characteristic of animals, homologous in those groups in which it is found and repeatedly lost in those groups that lack it; or it may have evolved repeatedly via convergent evolution as a similar adaptation to some consistent selective forces. This dilemma has not been resolved, and the origin of larvae remains the subject of intense debate (for review, see the opposing views of Raff [67] and Nielsen [68]).

The phylogenetic position and features of polyclad flatworms make them a valuable model with which to gain insight into the evolution of larval forms within the Spiralia. Although it has been argued that the existence of larvae in a single order of free-living platyhelminths points to its independent evolution in polyclads rather than repeated loss in most other flatworm clades [6], a basally branching position of the polyclads within platyhelminths allows it to be parsimoniously considered as a primitive character [8,69]. Polyclad flatworms can exhibit a range of developmental modes even within the same genus (Table 1): direct development with benthic juvenile worms, intermediate development with ciliated larvae metamorphosing within their eggshells and indirect development with pelagic swimming larvae that metamorphose postembryonically. These larvae have been classified into three main types: Kato’s larval features resemble those of a modified juvenile, whereas ciliary bands and cephalic ganglia found in Goette’s and Müller’s larvae have been thought by some to resemble those of the nemertean pilidium larva [70]. Others have suggested that the polyclad larva may be homologous to the trochophore larva [71].

The practical advantages and pioneering works on *M. crozieri*’s larva, together with the growing access to large-scale molecular, imaging and phylogenetic tools, will help to elucidate the evolution of larval forms and features. Evidence of homology of Müller’s larval characteristics with those of other spiralian larvae would hint at indirect development as the primitive platyhelminth and spiralian condition. On the other hand, the nonhomology of larval characteristics would suggest that similar larval forms can evolve repeatedly and independently.

### Evolution of the gut

Development of a through gut in metazoans has been a critical innovation that provides efficient food-processing. Despite this importance, its evolution is still surprisingly enigmatic. Within Eumetazoa, blind guts without a separate anus are found in ctenophores and cnidarians, whereas most bilaterians typically develop a through gut with two distinct openings. Some notable exceptions exist within bilaterians, however, as some species develop only a blind gut. This is the case for Xenacoelomorpha, Ophiuroidea, some Brachiopoda, some Rotifera, some Gnathostomulida and almost all Platyhelminthes [72]. Two scenarios can be envisioned to explain the presence of a blind gut in those animals: Either they retained a characteristic that was present in their stem group, or they secondarily lost an anus that was already present in their stem group.

The nested position of Platyhelminthes within spiralians (for example, [3]) suggests that a secondary loss of the anus in flatworms is the most parsimonious explanation. In support of this view, it has been suggested that a divergent developmental program could account for the absence of a through gut in Platyhelminthes. The most obvious example is found in polyclads whereby fourth-quartet macromeres (4A–4D), which give rise to endodermal structures in other spiralians, degenerate [10,11,71,73]. Also, many of the genes found expressed along the anteroposterior axis of bilaterian guts are not expressed in the gut or were lost in triclads [73]. Only one of the three ParaHox genes, *xlox*, is found in *Schmidtea mediterranea* and *Schmidtea polychroa* genomes, whereas *gsx* and *cdx*, as well as the T-box-containing gene *brachyury*, are missing [73]. The extent of such a loss is less pronounced in the Macrostomida [73] and in the polyclad *Discocelis tigrina*[74], which are more basally branched within the Platyhelminthes, indicating that these genes were secondarily lost in triclads and cannot be related to the lack of an anus in almost all Platyhelminthes.

Whilst our attempts to identify in the transcriptome of *M. crozieri* homologs of *brachyury* and *xlox* have been unsuccessful so far, we found homologs of *gsx* and *cdx* (Additional file 5: Figure S1, and Additional file 6). Saló and colleagues [74] reported the absence of a *gsx* homolog but the presence of an *xlox* homolog in the polyclad *Discocelis tigrina*, but its sequence has never been published and therefore could not be compared with the sequences from our transcriptome. It will be important in future studies to determine in *M. crozieri*’s embryos and juveniles the presence and the expression of these as well as other gut-related genes to better understand the evolutionary origin of the Platyhelminthes’ blind gut.

### Evolution of the nervous system and phototaxis

Many aspects of nervous system evolution remain elusive. One of them is the transition from the diffuse nervous system found in cnidarians to the centralised one found in most bilaterians [75,76]. Although some studies suggest that centralisation and complex patterning of the nervous system in adult animals predates the protostome–deuterostome split [77,78], others favour the idea that the urbilaterian possessed a far less complex nervous system [76,79].

The relatively simple nervous system of invertebrate ciliated larvae has been proposed to be informative regarding the evolution of the central nervous system [80], possibly recapitulating a transitional form *en route* to a complex adult nervous system. Conserved gene expression and immunoreactivity in the neurogenic region of these larvae [81-84] may hint at a common evolutionary history. However, the disappearance of the larval apical organ and ciliary band nerves during metamorphosis [80,85], and also the small number of markers or phyla investigated, make the evolutionary significance of those comparisons difficult to determine [86].

Whilst precise descriptions of the nervous system of *Macrostomum lignano*[2,87] and *Schmidtea mediterranea*[88] have already provided landmarks for juvenile and adult platyhelminth neuroanatomy, very little is known concerning platyhelminth larvae. In this respect, future studies on the nervous system of larval *M. crozieri* can provide an additional data set for determining the evolutionary significance of the nervous system of ciliated larvae.

Recent work on *Platynereis dumerilii* suggested that the phototactic behaviour of their larvae represent a paradigm for the evolution of the nervous system [49]. Phototaxis in *P. dumerilii* larvae relies on the presence of pigmented photoreceptors that connect directly to a ciliated locomotor cell of the prototrochal ring. In sponges and cnidarians, phototaxis relies on single cells, which have both light-detecting and ciliary locomotory functions, whereas several specialised cooperating cells are found in *P. dumerilii.* The latter could represent an early step in an evolutionary complexification of neural circuitry and visual systems [49].

*M. crozieri* larvae provide direct access to further testing of this hypothesis. In an assay similar to the one previously used for *P. dumerilii*[49], the polyclad larvae behaved positively phototactic (Figure 6M, Additional file 3 and Additional file 4; see also [13]), and they possess, like larvae of *Platynereis dumerilii*, rhabdomeric pigmented eyespots that develop closely associated with the bilateral cephalic ganglia [12], allowing close comparison between larvae of these two species.

The nervous system of *M. crozieri* larvae has been investigated using standard neuronal markers [12]. Expansion of this work with additional specific markers, such as neuropeptides, is warranted. Neuropeptides have an early evolutionary origin [89] and have been shown to be implicated in the control of swimming and settlement behaviour [90,91]. The rich repertoire of neuropeptides found in *Platynereis dumerilii*, together with a microscopic registration technique [92,93], have proven to be powerful tools to characterise and map individual neurons in a whole larva.

*M. crozieri* is especially amenable to similar approaches. The recently established transcriptome of *M. crozieri* allowed us to identify a number of conserved neuropeptide motifs, such as an AVRLIRLamide and a GVWSNDPWamide. Antibodies directed against the mature form of the neuropeptide show that distinct subsets of cells in *M. crozieri* are immunoreactive to these antibodies (Figure 7F and 7G; for a suitable staining protocol, see [12]). Determination of the extent of homology and specificity in development and nervous topology between *M. crozieri* and other spiralians will have repercussions on our understanding of neuronal evolution. The increased availability of neuropeptidomes in other larvae, such as the sea urchin [94,95], and two recent global analyses of neuropeptide evolution in Metazoa [96,97] should provide a solid comparison framework for all bilaterians.

## Conclusions

*M. crozieri*’s large size at maturity facilitates the extraction of hundreds of naked embryos from the adult and also makes the collection of individuals from the field easier. Adult worms can be kept in the laboratory without food for a considerable time and still produce eggs. The larvae can be kept alive in the laboratory for weeks, although raising them to metamorphosis has not been achieved to date.

An embryonic and larval transcriptome has been sequenced and assembled and is currently being analysed and complemented by full-genome sequencing. These resources are facilitating obtaining genes of interest for *in situ* probe synthesis, among others. We are in the process of developing a protocol for whole-mount *in situ* hybridization for *M. crozieri*. Whole-mount immunofluorescent staining works well [12] (Figure 7E through 7G), and we have produced polyclonal antibodies against some neuropeptides identified in the transcriptome (Figure 7F and 7G).

## Competing interests

The authors declare that they have no competing interests.

## Authors’ contributions

BE, FL, KR and MJT designed the experiments. BE, FL, JG, MJT and KR collected adult animals. BE, FL, JG, MJT and KR contributed pictures of adult animals. BE carried out regeneration experiments. KR collected material for transcriptomics and extracted RNA. MJT assembled the transcriptome. BE, BT, FL, JG, KR and MJT analysed the transcriptome. BE fixed larvae for scanning electron microscopy (SEM). GJ and JB made the SEM pictures. BE, GJ and MJT designed neuropeptide antibodies. BE did the immunostaining. BE, BT, FL, JG and KR conducted *in situ* hybridization trials. BE and FL recorded embryonic development and made larval differential interference contrast pictures. BE, FL and GJ did phototactic experiments and analysis. BE, BT, FL and JG prepared figures. BE and FL wrote the manuscript draft. All authors read, corrected and approved the final manuscript.

## Supplementary Material

Additional file 1 **Sperm transfer of adult** ***Maritigrella crozieri.***
Click here for file

Additional file 2 **Adult** ***Maritigrella crozieri*** **gliding under the water’s surface and then dropping to the bottom of the tank.** Click here for file

Additional file 3 **Phototactic behaviour of 2-day-old*Maritigrella crozieri*larvae in an embryo dish illuminated on the left side.** Larvae are swimming in a right-handed helical motion towards the light source. Click here for file

Additional file 4 **Trajectories of 2-day-old*Maritigrella crozieri*larvae swimming towards a light source situated on the left side.** Video is Additional file 3, in which a threshold filter was applied using Fiji software (http://www.fiji.sc). Coloured lines and dots were produced using the MTrackJ plugin [50] for Fiji and correspond to the trajectories of five selected larvae. Click here for file

Additional file 5: Figure S1 Alignment of the *Maritigrella crozieri* ParaHox genes predicted from the transcriptome. *M. crozieri*’s sequences are highlighted in bold. Sequences were aligned with other representative platyhelminth and metazoan sequences using Clustal Omega software (http://www.clustal.org/omega) [98] and visualised using Jalview software (http://www.jalview.org) [99] with colour code ClustalX. Only part of the alignment surrounding the conserved homeodomain is shown. The accession numbers for the sequences included in the alignment are Hs_CDX1 [Swiss-Prot:P47902], Hs_CDX2 [Swiss-Prot:Q99626], Sk_Caudal [Swiss-Prot:B5B3S6], Tc_caudal-1 [Swiss-Prot:D2A357], Tc_caudal-2 [Swiss-Prot:D2A356], Dt_Cdx [Swiss-Prot:Q9GP48], Pd_Cdx [Swiss-Prot:C7SB55], Ch_Cdx [Swiss-Prot:B9V2C5], Hs_PDX1 [Swiss-Prot:P52945], Dr_pdx1 [Swiss-Prot:Q6DC85], Sk_Lox2 [NCBI Refseq:XP_002741152.1], Sp_Xlox [Swiss-Prot:F1CDE7], Pd_Xlox [Swiss-Prot:C7SB60], Gv_Xlox [Swiss-Prot:D9IDZ2], Nv_Xlox/Cdx [Swiss-Prot:C7E1Y2], Hs_GSX1 [Swiss-Prot:Q9H4S2], Hs_GSX2 [Swiss-Prot:Q9BZM3], Dr_gsx1 [Swiss-Prot:Q5QHS3], Dr_gsx2 [Swiss-Prot:Q1RMA3], Pf_Gsx [Swiss-Prot:Q6T4Q6], Dm_Ind [Swiss-Prot:Q7KUL4], Sm_Gsx [NCBI Refseq:XP_002574409.1], Pd_Gsx [Swiss-Prot:C7SB47], Es_Gsx [Swiss-Prot:Q49QY0], Nv_GSX [Swiss-Prot:Q0ZRK1] and Ta_Gsx [Swiss-prot:B5LDS8]. Sequences from *Macrostomum lignano* were obtained by performing a BLAST analysis of its genome and transcriptome (http://www.macgenome.org/blast/index.html) using the ML100925 and MLRNA100918 assembly, respectively. The identification numbers of the *Macrostomum* sequences included in the alignment are Ml_Gsx1 [deg2520075501865], Ml_Gsx2 [deg2520075338729], Ml_Cdx [RNA918_2379] and Ml_Xlox [deg2520075475120). The sequences of the contigs corresponding to the *Maritigrella cdx* and *gsx* genes can be found in Additional file 1. The abbreviations for the species names in the alignment are as follows: Ct, *Clytia hemisphaerica*; Dm, *Drosophila melanogaster*; Dr, *Danio rerio*; Dt, *Discocelis tigrina*; Es, *Euprymna scolopes*; Gv, *Gibbula varia*; Hs, *Homo sapiens*; Mc, *Maritigrella crozieri*; Ml, *Macrostomum lignano*; Nv, *Nematostella vectensis*; Pd, *Platynereis dumerilii*; Pf, *Ptychodera flava*; Sk, *Saccoglossus kowalevskii*; Sm, *Schistosoma mansoni*; Sp, *Schmidtea polychroa*; Ta, *Trichoplax adhaerens*; Tc, *Tribolium castaneum*. Click here for file

Additional file 6 **Sequence of the contigs from*Maritigrella crozieri*’s transcriptome corresponding to the*cdx*and*gsx*genes.** Mc_Cdx contains a full coding sequence, whereas Mc_Gsx contains only a partial sequence. Click here for file
