# Supplementary material for: Put a tiger in your tank: the polyclad flatworm Maritigrella crozieri as a proposed model for evo-devo
Source: EvoDevo. 2013 Oct 9;4:29. doi: 10.1186/2041-9139-4-29 (PMC4124852; doi:10.1186/2041-9139-4-29)
Supplement: Additional file 5: Figure S1 — Alignment of the Maritigrella crozieri ParaHox genes predicted from the transcriptome. M. crozieri’s sequences are highlighted in bold. Sequences were aligned with other representative platyhelminth and metazoan sequences using Clustal Omega software (http://www.clustal.org/omega) [98] and visualised using Jalview software (http://www.jalview.org) [99] with colour code ClustalX. Only part of the alignment surrounding the conserved homeodomain is shown. The accession numbers for the sequences included in the alignment are Hs_CDX1 [Swiss-Prot:P47902], Hs_CDX2 [Swiss-Prot:Q99626], Sk_Caudal [Swiss-Prot:B5B3S6], Tc_caudal-1 [Swiss-Prot:D2A357], Tc_caudal-2 [Swiss-Prot:D2A356], Dt_Cdx [Swiss-Prot:Q9GP48], Pd_Cdx [Swiss-Prot:C7SB55], Ch_Cdx [Swiss-Prot:B9V2C5], Hs_PDX1 [Swiss-Prot:P52945], Dr_pdx1 [Swiss-Prot:Q6DC85], Sk_Lox2 [NCBI Refseq:XP_002741152.1], Sp_Xlox [Swiss-Prot:F1CDE7], Pd_Xlox [Swiss-Prot:C7SB60], Gv_Xlox [Swiss-Prot:D9IDZ2], Nv_Xlox/Cdx [Swiss-Prot:C7E1Y2], Hs_GSX1 [Swiss-Prot:Q9H4S2], Hs_GSX2 [Swiss-Prot:Q9BZM3], Dr_gsx1 [Swiss-Prot:Q5QHS3], Dr_gsx2 [Swiss-Prot:Q1RMA3], Pf_Gsx [Swiss-Prot:Q6T4Q6], Dm_Ind [Swiss-Prot:Q7KUL4], Sm_Gsx [NCBI Refseq:XP_002574409.1], Pd_Gsx [Swiss-Prot:C7SB47], Es_Gsx [Swiss-Prot:Q49QY0], Nv_GSX [Swiss-Prot:Q0ZRK1] and Ta_Gsx [Swiss-prot:B5LDS8]. Sequences from Macrostomum lignano were obtained by performing a BLAST analysis of its genome and transcriptome (http://www.macgenome.org/blast/index.html) using the ML100925 and MLRNA100918 assembly, respectively. The identification numbers of the Macrostomum sequences included in the alignment are Ml_Gsx1 [deg2520075501865], Ml_Gsx2 [deg2520075338729], Ml_Cdx [RNA918_2379] and Ml_Xlox [deg2520075475120). The sequences of the contigs corresponding to the Maritigrella cdx and gsx genes can be found in Additional file 1. The abbreviations for the species names in the alignment are as follows: Ct, Clytia hemisphaerica; Dm, Drosophila melanogaster; Dr, Danio rerio; Dt, Discocelis tigrina; [file 2041-9139-4-29-S5.pdf]

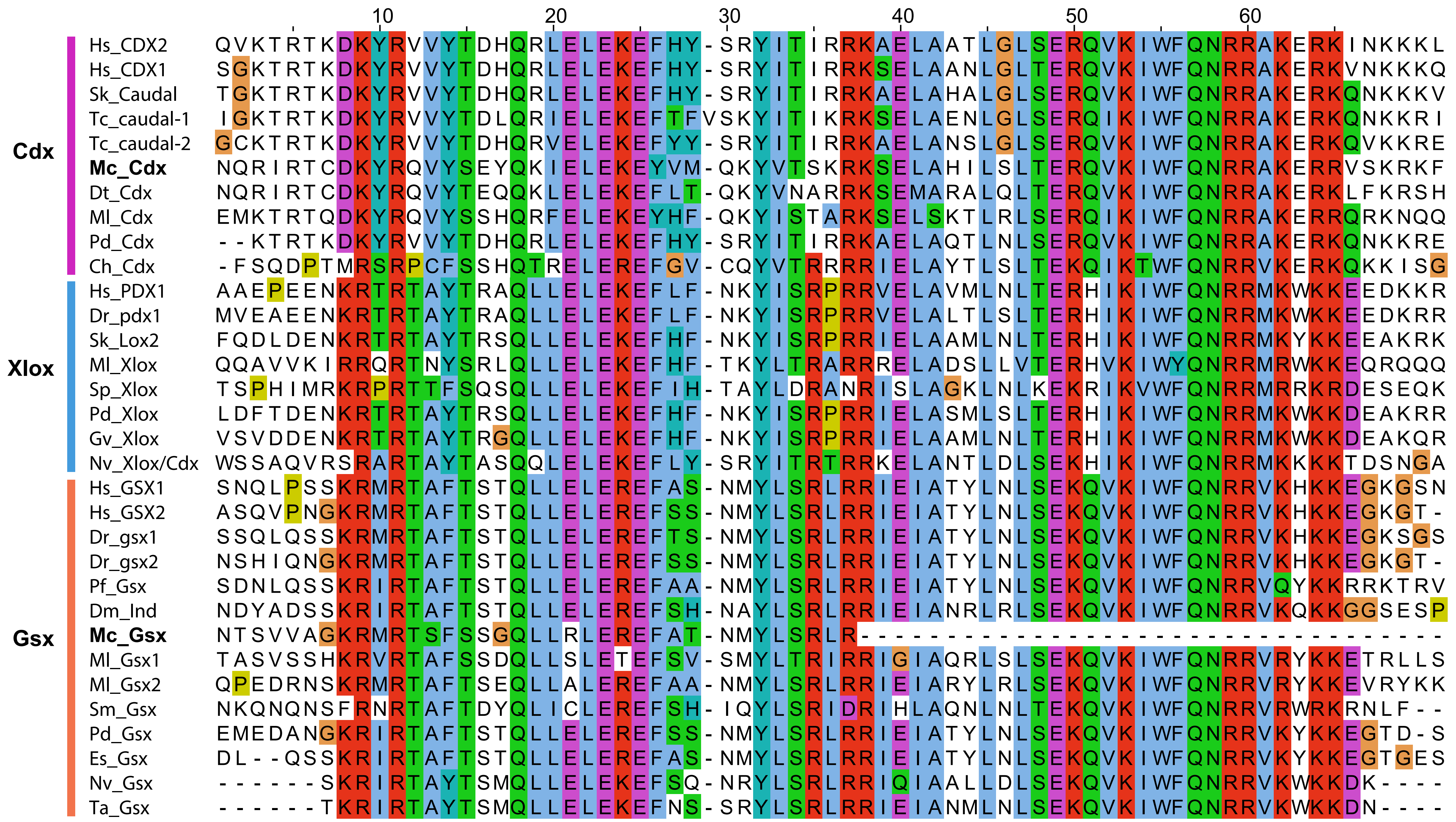

10 20 30 40 50 60
Cdx
Xlox
Gsx
Hs_CDX2 QVKTRTKDKYRVVYTDHQRLELEKEFHY-SRYITIRRKAELAATLGLSERQVKIWFQNRRAKERKINKKKL
Hs_CDX1 SGKTRTKDKYRVVYTDHQRLELEKEFHY-SRYITIRRKSELAANLGLTERQVKIWFQNRRAKERKVNKKKQ
Sk_Caudal TGKTRTKDKYRVVYTDHQRLELEKEFHY-SRYITIRRKAELAHALGLSERQVKIWFQNRRAKERKQNKKKV
Tc_caudal-1 IGKTRTKDKYRVVYTDLQRIELEKEFTFVSKYITIKRKSELAENLGLSERQIKIWFQNRRAKERKQNKKRI
Tc_caudal-2 GCKTRTKDKYRVVYTDHQRVELEKEFYY-SRYITIRRKAELANSLGLSERQVKIWFQNRRAKERKQVKKRE
Mc_Cdx NQRIRTCDKYRQVYSEYQKIELEKEYVM-QKYVTSKRKSELAHILSLTERQVKIWFQNRRAKERRVSKRKF
Dt_Cdx NQRIRTCDKYRQVYTEQQKLELEKEFLT-QKYVNARRKSEMARALQLTERQVKIWFQNRRAKERKLFKRSH
Ml_Cdx EMKTRTQDKYRQVYSSHQRFELEKEYHF-QKYISTARKSELSKTLRLSERQIKIWFQNRRAKERRQRKNQQ
Pd_Cdx --KTRTKDKYRVVYTDHQRLELEKEFHY-SRYITIRRKAELAQTLNLSERQVKIWFQNRRAKERKQNKKRE
Ch_Cdx -FSQDPTMRSRPCFSSHQTRELEREFGV-CQYVTRRRRIELAYTLSLTEKQIKTWFQNRRVKERKQKKISG
Hs_PDX1 AAEPEENKRTRTAYTRAQLLELEKEFLF-NKYISRPRRVELAVMLNLTERHIKIWFQNRRMKWKKEEDKKR
Dr_pdx1 MVEAEENKRTRTAYTRAQLLELEKEFLF-NKYISRPRRVELALTLSLTERHIKIWFQNRRMKWKKEEDKRR
Sk_Lox2 FQDLDENKRTRTAYTRSQLLELEKEFHF-NKYISRPRRIELAAMLNLTERHIKIWFQNRRMKYKKEEAKRK
Ml_Xlox QQAVVKIRRQRTNYSRLQLLELEKEFHF-TKYLTRARRRELADSLLVTERHVKIWYQNRRMRWKKEQRQQQ
Sp_Xlox TSPHIMRKRPRTTFSQSQLLELEKEFIH-TAYLDRANRISLAGKLNLKEKRIKVWFQNRRMRRKRDESEQK
Pd_Xlox LDFTDENKRTRTAYTRSQLLELEKEFHF-NKYISRPRRIELASMLSLTERHIKIWFQNRRMKWKKDEAKRR
Gv_Xlox VSVDDENKRTRTAYTRGQLLELEKEFHF-NKYISRPRRIELAAMLNLTERHIKIWFQNRRMKWKKDEAKQR
Nv_Xlox/Cdx WSSAQVRSRARTAYTASQQLELEKEFLY-SRYITRTRRKELANTLDLSEKHIKIWFQNRRMKKKKTDSNGA
Hs_GSX1 SNQLPSSKRMRTAFTSTQLLELEREFAS-NMYLSRLRRIEIATYLNLSEKQVKIWFQNRRVKHKKEGKGSN
Hs_GSX2 ASQVPNGKRMRTAFTSTQLLELEREFSS-NMYLSRLRRIEIATYLNLSEKQVKIWFQNRRVKHKKEGKGT-
Dr_gsx1 SSQLQSSKRMRTAFTSTQLLELEREFTS-NMYLSRLRRIEIATYLNLSEKQVKIWFQNRRVKHKKEGKSGS
Dr_gsx2 NSHIQNGKRMRTAFTSTQLLELEREFSS-NMYLSRLRRIEIATYLNLSEKQVKIWFQNRRVKHKKEGKGT-
Pf_Gsx SDNLQSSKRIRTAFTSTQLLELEREFAA-NMYLSRLRRIEIATYLNLSEKQVKIWFQNRRVQYKKRRKTRV
Dm_Ind NDYADSSKRIRTAFTSTQLLELEREFSH-NAYLSRLRRIEIANRLRLSEKQVKIWFQNRRVKQKKGGSESP
Mc_Gsx NTSVVAGKRMRTSFSSGQLLRLEREFAT-NMYLSRLR----------------------------------
Ml_Gsx1 TASVSSHKRVRTAFSSDQLLSLETEFSV-SMYLTRIRRIGIAQRLSLSEKQVKIWFQNRRVRYKKETRLLS
Ml_Gsx2 QPEDRNSKRMRTAFTSEQLLALEREFAA-NMYLSRLRRIEIARYLRLSEKQVKIWFQNRRVRYKKEVRYKK
Sm_Gsx NKQNQNSFRNRTAFTDYQLICLEREFSH-IQYLSRIDRIHLAQNLNLTEKQVKIWFQNRRVRWRKRNLF--
Pd_Gsx EMEDANGKRIRTAFTSTQLLELEREFSS-NMYLSRLRRIEIATYLNLSEKQVKIWFQNRRVKYKKEGTD-S
Es_Gsx DL--QSSKRIRTAFTSTQLLELEREFAS-NMYLSRLRRIEIATYLNLSEKQVKIWFQNRRVKYKKEGTGES
Nv_Gsx -------SKRIRTAYTSMQLLELEKEFSQ-NRYLSRLRRIQIAALLDLSEKQVKIWFQNRRVKWKKDK----
Ta_Gsx -------TKRIRTAYTSMQLLELEKEFNS-SRYLSRLRRIEIANMLNLSEKQVKIWFQNRRVKWKKDN----
