# Supplementary material for: Put a tiger in your tank: the polyclad flatworm Maritigrella crozieri as a proposed model for evo-devo
Source: EvoDevo. 2013 Oct 9;4:29. doi: 10.1186/2041-9139-4-29 (PMC4124852; doi:10.1186/2041-9139-4-29)
Supplement: Additional file 6 — Sequence of the contigs fromMaritigrella crozieri’s transcriptome corresponding to thecdxandgsxgenes. Mc_Cdx contains a full coding sequence, whereas Mc_Gsx contains only a partial sequence. [file 2041-9139-4-29-S6.rtf]

>Mc_GsxAATCATTTATGGTGGAGGACATAGTTGGGCATAATGATCAGAGAAACTTATTATTGGGGTCCGAGATCACTACAGTTCATCCACAGACCACTGCTTACACACTTTACCCTTCCGGACTTTATTCCTGTTGTGGAACCGGCTCCTGTACGTCGTCGAATTTGACGGCGAGATGTTGGATTCATCCATCAGCATTCTCTCCCACATTCCCAGTTGGAGCCGATCCGTTTCAACGGGTCTATGGCCTTCCAAGATCTGAGACCGACAGAATGGGCCATTCGACATACTGCTCTCGTCACCCACTGGAAGAATCATTGCAAATGGATGCCTCGCTATCATTGCACTCGCAGAATGGAGATTCGAATACCTCTGTCGTGGCTGGGAAACGAATGAGAACCTCCTTCTCCAGCGGTCAACTCCTTCGATTGGAACGCGAATTTGCAACAAACATGTATCTCTCCAGGCTTCG>Mc_CdxGCGAAATATTATAAGTTTTTTGTACAATCTTTCTATTTACAAAGATATACTAGTCAAAGGCCGAGCATCGATTTCATTGGCTAAAATGACGTGGACAGCTGATCTATCGATGAGTCATCCAATGGAGAAGTCAATAAATCCGTACTCATAAAAGAGAATTTTCGTTTACTGACACGACGTTCCTTCGCTCTTCGATTTTGAAACCATATCTTCACCTGCCGTTCTGTCAAGCTGAGAATATGGGCCAATTCACTCTTTCTCTTTGACGTCACATATTTTTGCATCACATACTCTTTCTCCAGCTCTATTTTCTGATATTCTGAATATACTTGGCGATATTTATCACACGTTCGGATCCGTTGATTTTTCGTACTTGCACGGGGTCTAATTGCTGCAGGCATCGTTGGCGTGTTCGCTAATACGCAATCAGGCATTTGAACTGAGGAGGGTGAAGTGTTGCTTGGGTAATTCCCCGGCAATTGTTCTGAGTACCAGCACTCATTCCCTATAGAATATGTAGCAGCGTGGTAGCAGTGAGGGCATTGACAAGGCACTGGATACCGCGTGGACGGGAACTGATACATGTTATAGCGTTCCATCGGATCAGGTTGATGAGACTGCTGTTGAGTTCGAAGTGATGGGAAGTCCTTTGAGGCGTGCAGTTGTGGCTGTTCTGAGAACGGTTGATGGCGTGTTTGAGGGAAATGTTGAGGCGGTCCGGATGGTAGTGAAGGGTCGCTCATGTGGACGGGAGAGTGAGATGAGACGTGTGCCGTCATGGGATGTCTCAGAGCACTAACAGTAGGTAACTGTGGAGGCTGCGGTGTGGCGAGGTGTGTCGGAGAGGTTGTTGGGAGCAGGCAAGGCAAGGTCGACGCAATGGAGGAAAAGTGGTTCCTTTGATGTGCGCCGCCGTACAATTCACCATTGACGAGTGCATGTTCTATCGGCATGGCCATGGGAAGCCTAACGATGGTCTCTCCTGGCTTTATCTTTCTTGTCGAGTTGCCGTCAATTCGATGACTGTGCGGTTAGAGTAGTGTCGCCTCAGAAATTGCACGTTCGTATGCGTGTGGCAGATTGTGTTTGTAACGACTCCTAACGGCCCTTAGACTTTTACTCCCCCAAATGCCGAGATCGGAAGAG
